# A highly penetrant *ACTA2* mutation of thoracic aortic disease

**DOI:** 10.1186/s13019-023-02420-0

**Published:** 2023-12-04

**Authors:** Christopher M. Bobba, Ryan Azarrafiy, John R. Spratt, Jill Hendrickson, Tomas D. Martin, George J. Arnaoutakis, Eric I. Jeng, Thomas M. Beaver

**Affiliations:** 1 Division of Cardiovascular Surgery, Department of Surgery, University of Florida, 1600 SW Archer Road, Gainesville, FL 32601 USA; 2 UF Health Aortic Disease Center, University of Florida, Gainesville, FL USA

**Keywords:** Aorta, Aortic arch, Aortic operation, Aortic genetics

## Abstract

**Background:**

The role of *ACTA2* mutations in Familial Aortic Disease has been increasingly recognized. We describe a highly penetrant variant (R118Q) in a family with aortic disease.

**Case report:**

A patient presented to us for elective repair of an ascending aortic aneurysm with a family history of his mother expiring after aortic dissection. Genetic testing revealed he was a heterozygous carrier of the *ACTA2* missense mutation R118Q. Subsequently, all living family members were tested for this variant and a full medical history was obtained to compile a family tree for the variant and penetrance of an aortic event (defined as lifetime occurrence of aortic surgery / dissection). In total 9 family members were identified and underwent genetic testing with 7/9 showing presence of the *ACTA2* R118Q mutation or an aortic event. All patients over the age of 50 (n = 4) had an aortic event. Those events occurred at ages 54, 55, 60, and 62 (mean event at 57.8 ± 3.9 years). Three family members with the variant under the age of 40 have not had an aortic event and most are undergoing regular aortic surveillance via CT scan.

**Conclusions:**

Existing studies of known *ACTA2* mutations describe a 76% aortic event rate by 85 years old. The R118Q missense mutation is a less common *ACTA2* variant, estimated to be found in about 5% of patients with known mutations. Prior studies have predicted the R118Q mutation to have a slightly decreased risk of aortic events compared to other *ACTA2* mutations. In this family, however, we demonstrate 100% penetrance of aortic disease above age 50. In today’s era of excellent outcomes in elective aortic surgery, our team aggressively offers elective repair. We advocate for strict aortic surveillance for patients with this variant and would consider elective aortic replacement at 4.5 cm, or at an even smaller diameter in patients with a strong family history of dissection who are identified with this mutation.

**Supplementary Information:**

The online version contains supplementary material available at 10.1186/s13019-023-02420-0.

## Background

Some connective tissue disorders (CTD), including Marfan’s syndrome, vascular Ehlers-Danlos syndrome, and Loeys-Dietz syndrome, carry increased risk of aortic aneurysm formation and subsequent dissection. Current guidelines recommend surgical aortic replacement at smaller aortic diameters in CTD patients compared to non-CTD patients [1]. The proliferation of genetic testing in aortic disease has identified numerous discrete mutations associated with the formation of familial non-syndromic aortic aneurysms, which place patients at increased risk of aortic catastrophe. In this report we characterize a heterozygous mutation of the actin alpha 2 (*ACTA2*) gene thought to contribute to familial thoracic aortic aneurysms (OMIM ID 611788). Based on This variant, R118Q, is associated with a high, age-dependent penetrance of aortic disease.

## Case report

A 60-year old male was referred for evaluation of an ascending aortic aneurysm following several months of intermittent chest pain. CT angiography revealed a 4.8 cm aortic root and moderate aortic insufficiency was seen on transthoracic echocardiography. Family history was notable for a mother and brother who died from acute aortic dissection and a second brother who underwent emergent repair of a type A dissection. Due to his symptoms and strong family history, elective aortic repair was recommended to mitigate the risk of rupture/dissection and progression of aortic valved disease. The patient underwent aortic root replacement using a bioprosthetic composite valved conduit in a modified Bentall fashion. The patient tolerated the procedure well and was discharged home on postoperative day 4 following uneventful inpatient convalescence.

**Fig. 1:**
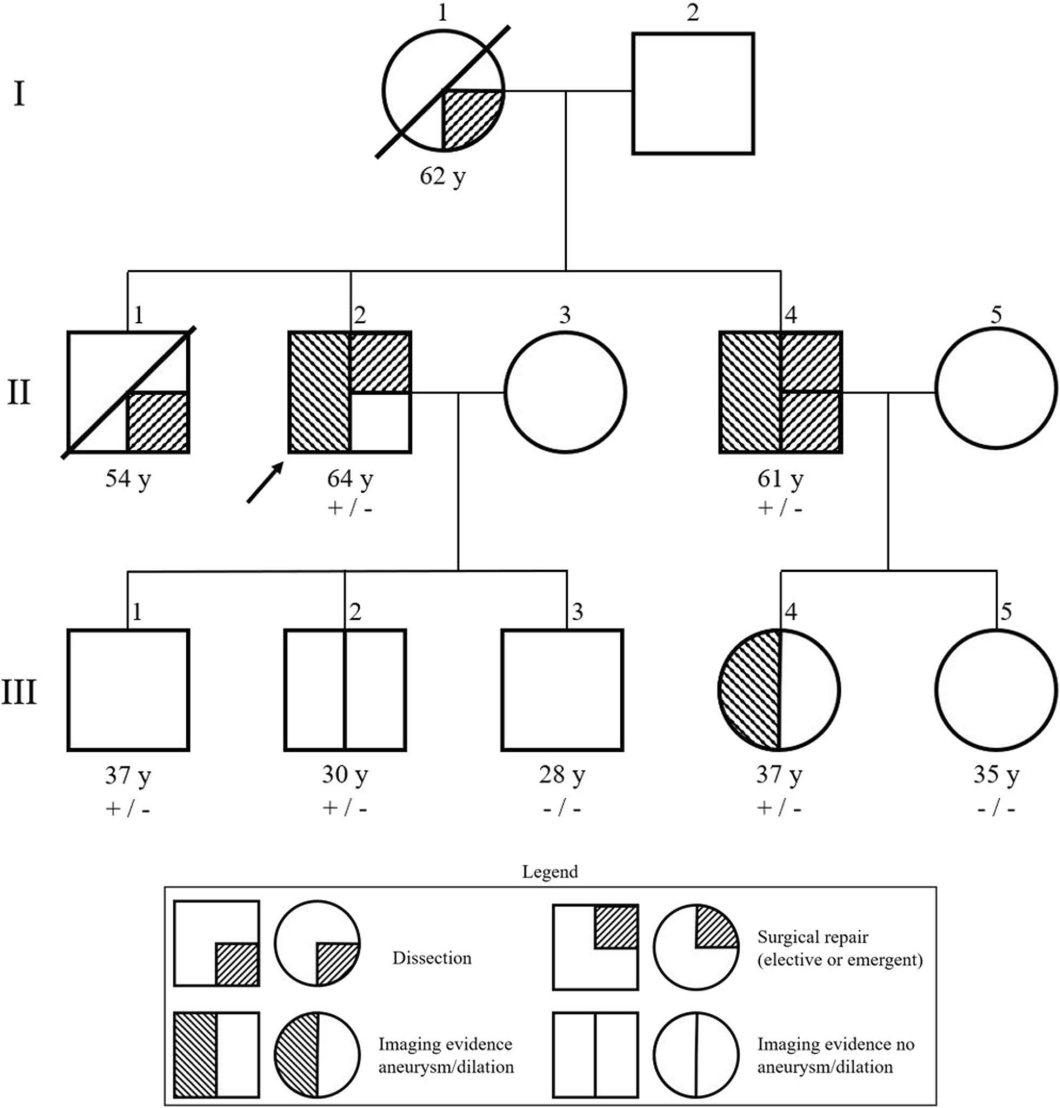
Family pedigree. The arrow indicates the proband (II-2). Plus signs (+) and minus signs (−) indicate presence and absence, respectively, of R118Q variant of the ACTA2 gene. Slashed line indicates death at the age indicated. Proband son (III-1) has undergone genetic testing but has declined CT surveillance for aneurysmal disease. Proband brother (II-4) underwent emergent repair for dissection at 60y

Given his strong family history, genetics consultation was sought and genetic testing was performed on the proband. A commercially available Invitae panel was used to identify a potential mutation. This revealed that he was a heterozygous carrier of the *ACTA2* missense mutation R118Q. All living family members were contacted and all agreed to be tested for this variant, allowing construction of a pedigree for the variant and penetrance of an aortic event (Fig. 1). An aortic event was defined as lifetime occurrence of aortic surgery or dissection. 7 family members underwent genetic testing and 5/7 harbored the variant. Of those 5: both members over the age of 50 had an aortic event (II-4 experienced a dissection with emergent repair and the proband II-2 underwent elective repair for large aneurysm), one member (III-4) has known aortic dilation and is monitored with regular surveillance CT angiography, one has imaging with normal aortic diameter (III-2, 3.8 cm ascending aorta), and one has declined imaging surveillance (III-1). Notably, all descendants of I-1 over the age of 50 had an aortic event. Regardless of genetic presence of genetic mutation, all descendants of I-1 over the age of 50 (n = 4) had an aortic event (mean age at event 57.8 ± 3.9 years).

## Discussion and conclusions

The *ACTA2* gene codes for a smooth muscle-specific isoform of α-actin and is involved in smooth muscle contraction. Mutations in this gene are the most frequently encountered non-syndromic cause of familial thoracic aortic disease, and estimated to encompass 14% of those with this disease [2]. A variety of *ACTA2* mutations have been characterized as pathogenic. The overall penetrance of an aortic event with any *ACTA2* mutation was observed to be 48%, with an estimated 75% risk of aortic event by 85 years of age. The specific R118Q missense mutation identified in this family is estimated to comprise about 5% of all *ACTA2* mutations with an estimated 76% lifetime risk of aortic event by 85 years of age [3]. Although published estimates of risk profiles for specific variants are hindered by small patient cohort sizes, R118Q is also estimated to confer a lower risk of aortic event than other characterized mutations (i.e. R179). Though we do not have genetic testing for 2/4 family members who passed away from dissection, in this family we observed a 100% penetrance of aortic events in family members > 50 years of age.This suggests a higher risk and age dependent penetrance with this variant than previously estimated (Additional file 1).

In the current era of excellent outcomes in elective aortic surgery, we advocate an aggressive approach to elective repair in patients at elevated risk for aortic events. We further advocate strict aortic surveillance in patients with this and other high-risk genetic variants. The decision for repair should account for family history (including age of dissection of other family members), patient preferences, and concomitant cardiovascular pathology [4]. Threshold aneurysm diameter for repair in these patients remains controversial but repair at diameters ≥ 4.0–4.5 cm should be strongly considered in otherwise healthy patients with a strong family history of dissection [5, 6]. At our experienced center we advocate replacement at 4.5 cm in patients with an *ACTA2* mutation and a strong family history of dissection. As these disorders become increasingly recognized and families followed with imaging surveillance with elective aneurysmal repair when indicated, we hope for a reduction in morbidity and mortality in these families, We also advocate for strict surveillance and genetic testing in all patients with a family history of aortic dissection.

### Supplementary Information

**Additional file 1.** Supplementary Figures.

## Data Availability

Not applicable.

## Abbreviations

CTD: Connective tissue disorder
*ACTA2*: Acta alphin 2 gene

## References

[1] Falk V, Baumgartner H, Bax JJ, De Bonis M, Hamm C, Holm PJ (2017). 2017 ESC/EACTS guidelines for the management of valvular heart disease. Eur J Cardiothorac Surg.

[2] Guo DC, Pannu H, Tran-Fadulu V, Papke CL, Yu RK, Avidan N (2007). Mutations in smooth muscle alpha-actin (ACTA2) lead to thoracic aortic aneurysms and dissections. Nat Genet.

[3] Regalado ES, Guo DC, Prakash S, Bensend TA, Flynn K, Estrera A (2015). Aortic disease presentation and outcome associated with ACTA2 mutations. Circ Cardiovasc Genet.

[4] Chou AS, Ma WG, Mok SC, Ziganshin BA, Peterss S, Rizzo JA, Tranquilli M (2017). Do familial aortic dissections tend to occur at the same age?. Ann Thorac Surg.

[5] Ziganshin BA, Zafar MA, Elefteriades JA (2019). Descending threshold for ascending aortic aneurysmectomy: is it time for a "left-shift" in guidelines?. J Thorac Cardiovasc Surg.

[6] Isselbacher EM, Preventza O, Hamilton Black J, Augoustides JG, Beck AW, Bolen MA (2022). ACC/AHA guideline for the diagnosis and management of aortic disease: a report of the American Heart Association/American College of Cardiology joint committee on clinical practice guidelines. Circulation.

