# Supplementary figures and images for: A highly penetrant ACTA2 mutation of thoracic aortic disease
Source: J Cardiothorac Surg. 2023 Dec 4;18:352. doi: 10.1186/s13019-023-02420-0 (PMC10694883; doi:10.1186/s13019-023-02420-0)

## Slide 1
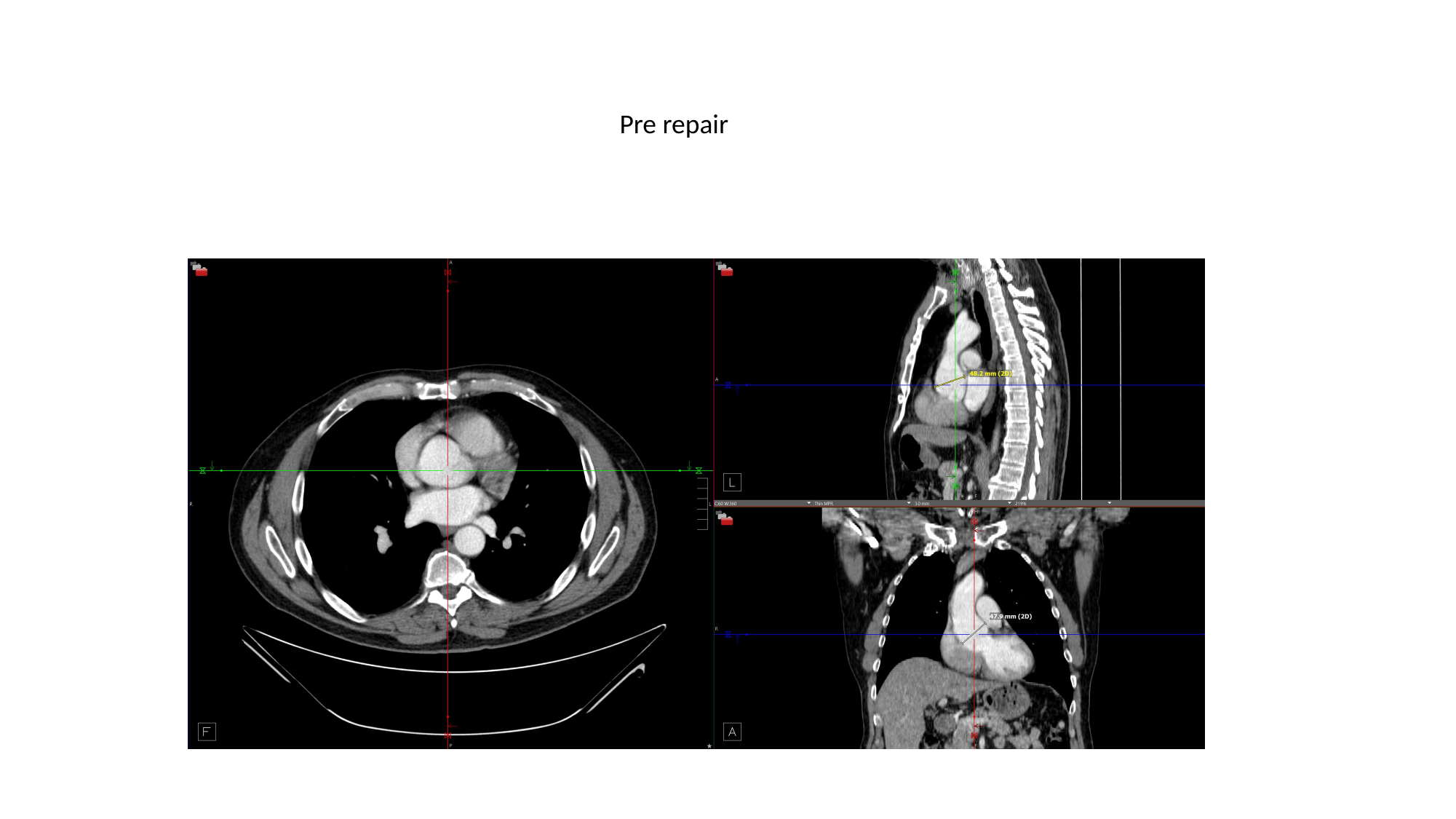

Pre repair

## Slide 2
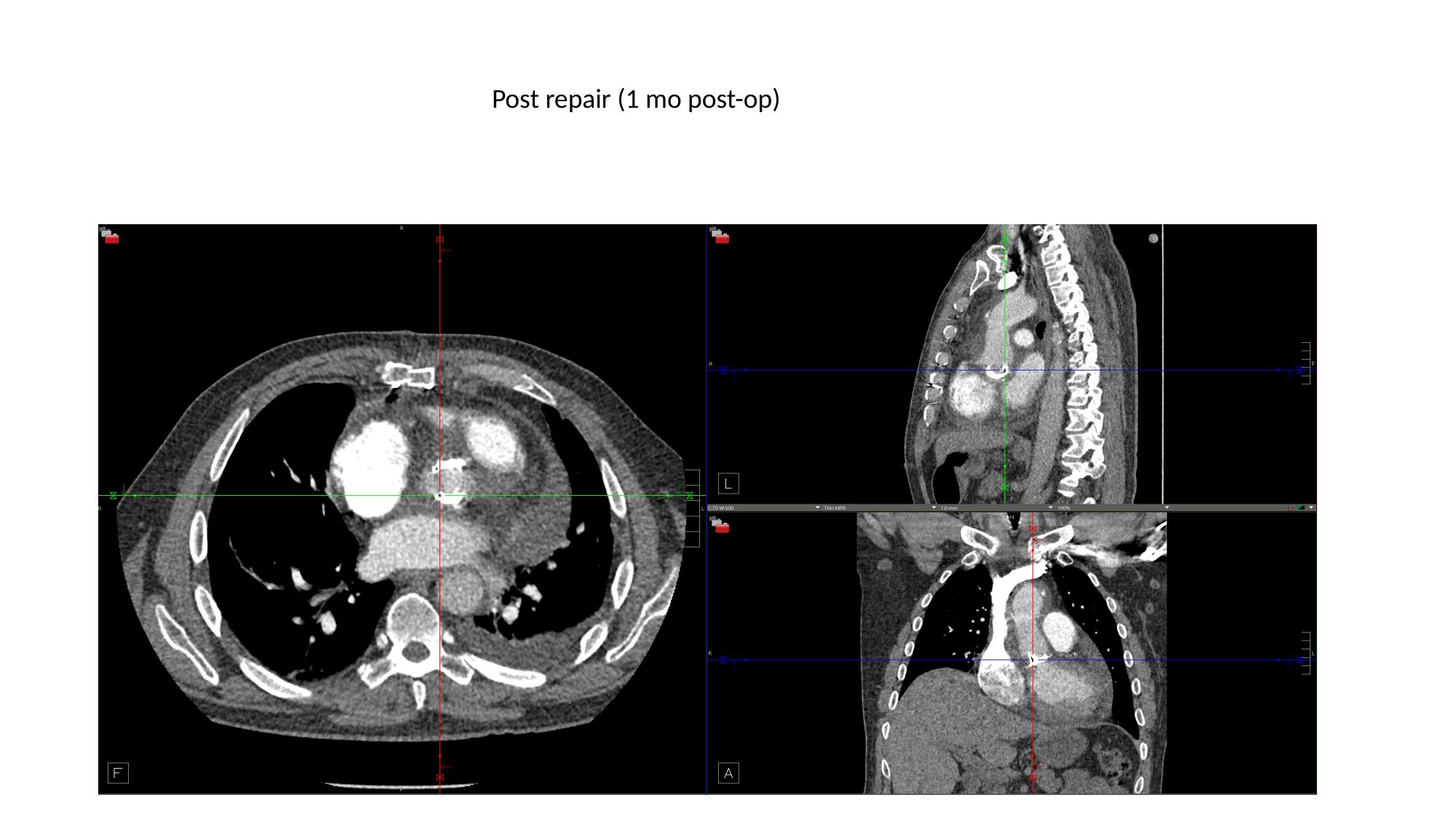

Post repair (1 mo post-op)

Supplement: Supplementary file 1 — Additional file 1. Supplementary Figures. [file 13019_2023_2420_MOESM1_ESM.pptx]
